# (4-Cyano­phen­yl)methyl­ene diacetate

**DOI:** 10.1107/S1600536808012543

**Published:** 2008-05-03

**Authors:** Jie Xiao, Hong Zhao

**Affiliations:** a Ordered Matter Science Research Center, College of Chemistry and Chemical Engineering, Southeast University, Nanjing 210096, People’s Republic of China

## Abstract

In the title mol­ecule, C_12_H_11_NO_4_, the two acetyl groups are inclined by 71.3 (1) and 46.2 (1)° to the benzene ring. In the crystal structure, mol­ecules are linked into a chain along the *c* axis by C—H⋯O hydrogen bonds.

## Related literature

For general background on nitrile compounds, see: Jin *et al.* (1994 ▶); Radl *et al.* (2000 ▶). For a related structure, see: Fu & Zhao (2007 ▶).
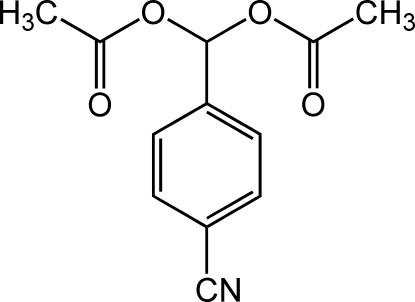


## Experimental

### 

#### Crystal data

C_12_H_11_NO_4_
*M* *_r_* = 233.22
Monoclinic, 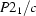

*a* = 8.1389 (15) Å
*b* = 20.919 (3) Å
*c* = 7.7748 (10) Å
β = 115.531 (7)°
*V* = 1194.5 (3) Å^3^
*Z* = 4
Mo *K*α radiation
μ = 0.10 mm^−1^
*T* = 293 (2) K
0.35 × 0.30 × 0.30 mm

#### Data collection

Rigaku Mercury2 diffractometer
Absorption correction: multi-scan (*CrystalClear*; Rigaku, 2005 ▶) *T*_min_ = 0.951, *T*_max_ = 0.968
11568 measured reflections
2723 independent reflections
2096 reflections with *I* > 2σ(*I*)
*R*_int_ = 0.035

#### Refinement

*R*[*F* ^2^ > 2σ(*F* ^2^)] = 0.059
*wR*(*F* ^2^) = 0.163
*S* = 1.11
2723 reflections
156 parameters
H-atom parameters constrained
Δρ_max_ = 0.20 e Å^−3^
Δρ_min_ = −0.17 e Å^−3^

### 

Data collection: *CrystalClear* (Rigaku, 2005 ▶); cell refinement: *CrystalClear*; data reduction: *CrystalClear*; program(s) used to solve structure: *SHELXS97* (Sheldrick, 2008 ▶); program(s) used to refine structure: *SHELXL97* (Sheldrick, 2008 ▶); molecular graphics: *SHELXTL* (Sheldrick, 2008 ▶); software used to prepare material for publication: *SHELXL97*.

## Supplementary Material

Crystal structure: contains datablocks I, global. DOI: 10.1107/S1600536808012543/ci2587sup1.cif

Structure factors: contains datablocks I. DOI: 10.1107/S1600536808012543/ci2587Isup2.hkl

Additional supplementary materials:  crystallographic information; 3D view; checkCIF report

## Figures and Tables

**Table 1:** Hydrogen-bond geometry (Å, °)

*D*—H⋯*A*	*D*—H	H⋯*A*	*D*⋯*A*	*D*—H⋯*A*
C1—H1⋯O2^i^	0.98	2.56	3.351 (3)	137

Symmetry code: (i) 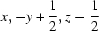.

**Fig. 1.**
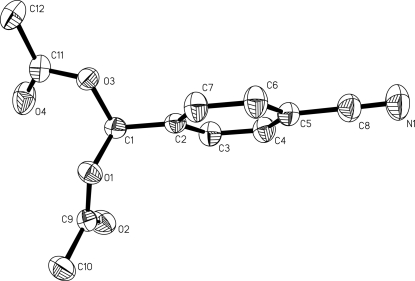
The molecular structure of the title compound, showing the atomic numbering scheme. Displacement ellipsoids are drawn at the 50% probability level.

## supplementary crystallographic information

### Comment

Nitrile compounds are used extensively in the chemical industry. They are discharged into the environment in industrial waste water, agricultural chemicals, *etc*. Nitrile derivatives are important materials in the synthesis of some heterocyclic molecules (Radl *et al.*, 2000), and they have been used as starting materials for phthalocyanines (Jin *et al.*, 1994). Recently, we have reported the crystal structure of a benzonitrile compound (Fu *et al.*, 2007). The title compound was unexpectedly obtained during our work on the nitrile compounds. Herein we report its crystal structure.

The bond lengths and angles have normal values. The O1/O2/C9/C10 and O3/O4/C11/C12 planes form dihedral angles of 71.3 (1)° and 46.2 (1)°, respectively, with the C2—C7 plane. The molecules are linked into a chain along the *c* axis by C—H···O hydrogen bonds (Table 1).

### Experimental

4-Formylbenzonitrile (0.262 mg, 2 mmol) was dissolved in acetic anhydride (5 ml) and heated under reflux for 3 h. The mixture was cooled to room temperature and the solution was filtered. The solvent was removed under vacuum from the filtrate to obtain a white precipitate of the title compound (yield 88%). Colourless crystals suitable for X-ray diffraction were obtained by slow evaporation of an ethanol (15 ml) solution of the compound (100 mg) after 4 d.

### Refinement

H atoms were positioned geometrically and refined using a riding model, with C—H = 0.93–0.98 Å and *U*_iso_(H) = 1.2–1.5*U*_eq_(C).

### Crystal data

**Table:**

C_12_H_11_NO_4_	*F*_000_ = 488
*M**_r_* = 233.22	*D*_x_ = 1.297 Mg m^−^^3^
Monoclinic, *P*2_1_/*c*	Mo *K*α radiation λ = 0.71073 Å
Hall symbol: -P 2ybc	Cell parameters from 2461 reflections
*a* = 8.1389 (15) Å	θ = 3.1–27.5º
*b* = 20.919 (3) Å	µ = 0.10 mm^−^^1^
*c* = 7.7748 (10) Å	*T* = 293 (2) K
β = 115.531 (7)º	Block, colourless
*V* = 1194.5 (3) Å^3^	0.35 × 0.30 × 0.30 mm
*Z* = 4	

### Data collection

**Table:**

Rigaku Mercury2 diffractometer	2723 independent reflections
Radiation source: fine-focus sealed tube	2096 reflections with *I* > 2σ(*I*)
Monochromator: graphite	*R*_int_ = 0.035
Detector resolution: 13.6612 pixels mm^-1^	θ_max_ = 27.5º
*T* = 293(2) K	θ_min_ = 3.1º
ω scans	*h* = −10→10
Absorption correction: multi-scan(CrystalClear; Rigaku, 2005)	*k* = −27→27
*T*_min_ = 0.951, *T*_max_ = 0.968	*l* = −10→10
11568 measured reflections	

### Refinement

**Table:**

Refinement on *F*^2^	Secondary atom site location: difference Fourier map
Least-squares matrix: full	Hydrogen site location: inferred from neighbouring sites
*R*[*F*^2^ > 2σ(*F*^2^)] = 0.060	H-atom parameters constrained
*wR*(*F*^2^) = 0.163	*w* = 1/[σ^2^(*F*_o_^2^) + (0.0727*P*)^2^ + 0.2026*P*] where *P* = (*F*_o_^2^ + 2*F*_c_^2^)/3
*S* = 1.11	(Δ/σ)_max_ = 0.001
2723 reflections	Δρ_max_ = 0.20 e Å^−^^3^
156 parameters	Δρ_min_ = −0.17 e Å^−^^3^
Primary atom site location: structure-invariant direct methods	Extinction correction: none

### Special details

**Table:**

Geometry. All e.s.d.'s (except the e.s.d. in the dihedral angle between two l.s. planes) are estimated using the full covariance matrix. The cell e.s.d.'s are taken into account individually in the estimation of e.s.d.'s in distances, angles and torsion angles; correlations between e.s.d.'s in cell parameters are only used when they are defined by crystal symmetry. An approximate (isotropic) treatment of cell e.s.d.'s is used for estimating e.s.d.'s involving l.s. planes.
Refinement. Refinement of *F*^2^ against ALL reflections. The weighted *R*-factor *wR* and goodness of fit *S* are based on *F*^2^, conventional *R*-factors *R* are based on *F*, with *F* set to zero for negative *F*^2^. The threshold expression of *F*^2^ > σ(*F*^2^) is used only for calculating *R*-factors(gt) *etc*. and is not relevant to the choice of reflections for refinement. *R*-factors based on *F*^2^ are statistically about twice as large as those based on *F*, and *R*- factors based on ALL data will be even larger.

### Fractional atomic coordinates and isotropic or equivalent isotropic displacement parameters (Å^2^)

**Table:**

	*x*	*y*	*z*	*U*_iso_*/*U*_eq_	
C1	0.4962 (2)	0.34433 (9)	0.7629 (3)	0.0477 (4)	
H1	0.5242	0.2985	0.7757	0.057*	
C2	0.6526 (2)	0.38246 (9)	0.7624 (2)	0.0462 (4)	
C3	0.8165 (3)	0.35208 (10)	0.8026 (3)	0.0586 (5)	
H3	0.8287	0.3086	0.8300	0.070*	
C4	0.9621 (3)	0.38610 (11)	0.8021 (3)	0.0626 (5)	
H4	1.0722	0.3656	0.8301	0.075*	
C5	0.9433 (3)	0.45058 (10)	0.7598 (3)	0.0565 (5)	
C6	0.7805 (3)	0.48137 (11)	0.7208 (4)	0.0693 (6)	
H6	0.7685	0.5249	0.6936	0.083*	
C7	0.6354 (3)	0.44710 (10)	0.7223 (3)	0.0627 (5)	
H7	0.5260	0.4677	0.6962	0.075*	
C8	1.0936 (3)	0.48634 (12)	0.7541 (4)	0.0701 (6)	
C9	0.5275 (3)	0.33307 (10)	1.0797 (3)	0.0535 (5)	
C10	0.4826 (4)	0.36266 (13)	1.2275 (4)	0.0779 (7)	
H10A	0.5070	0.3327	1.3292	0.117*	
H10B	0.3561	0.3742	1.1723	0.117*	
H10C	0.5557	0.4002	1.2768	0.117*	
C11	0.2023 (3)	0.31319 (10)	0.5370 (4)	0.0608 (5)	
C12	0.0457 (3)	0.33230 (13)	0.3577 (4)	0.0799 (7)	
H12A	−0.0238	0.2951	0.2961	0.120*	
H12B	0.0895	0.3526	0.2747	0.120*	
H12C	−0.0300	0.3616	0.3860	0.120*	
N1	1.2104 (3)	0.51476 (12)	0.7475 (4)	0.0967 (8)	
O1	0.45266 (18)	0.36634 (6)	0.91409 (19)	0.0536 (4)	
O2	0.6196 (2)	0.28659 (9)	1.0993 (2)	0.0757 (5)	
O3	0.33842 (17)	0.35734 (6)	0.59147 (18)	0.0545 (4)	
O4	0.2134 (2)	0.26588 (8)	0.6276 (3)	0.0905 (6)	

### Atomic displacement parameters (Å^2^)

**Table:**

	*U*^11^	*U*^22^	*U*^33^	*U*^12^	*U*^13^	*U*^23^
C1	0.0498 (10)	0.0476 (9)	0.0466 (9)	0.0048 (8)	0.0217 (8)	0.0008 (7)
C2	0.0465 (9)	0.0503 (10)	0.0406 (9)	0.0022 (8)	0.0178 (7)	−0.0018 (7)
C3	0.0548 (11)	0.0518 (11)	0.0689 (13)	0.0068 (9)	0.0266 (10)	0.0037 (9)
C4	0.0480 (11)	0.0687 (13)	0.0728 (14)	0.0092 (9)	0.0277 (10)	0.0020 (10)
C5	0.0496 (10)	0.0654 (12)	0.0553 (11)	−0.0037 (9)	0.0234 (9)	−0.0012 (9)
C6	0.0612 (13)	0.0515 (11)	0.0986 (18)	−0.0007 (10)	0.0377 (13)	0.0069 (11)
C7	0.0524 (11)	0.0526 (11)	0.0866 (15)	0.0078 (9)	0.0333 (11)	0.0069 (10)
C8	0.0574 (13)	0.0760 (15)	0.0783 (16)	−0.0037 (11)	0.0304 (11)	0.0028 (12)
C9	0.0505 (10)	0.0609 (12)	0.0505 (11)	−0.0104 (9)	0.0230 (9)	0.0027 (9)
C10	0.0912 (17)	0.0936 (18)	0.0642 (14)	−0.0232 (14)	0.0478 (13)	−0.0149 (12)
C11	0.0513 (11)	0.0548 (11)	0.0786 (14)	−0.0018 (9)	0.0303 (10)	−0.0020 (10)
C12	0.0525 (12)	0.0849 (16)	0.0844 (17)	−0.0072 (11)	0.0126 (11)	−0.0059 (13)
N1	0.0668 (14)	0.0997 (17)	0.129 (2)	−0.0114 (12)	0.0470 (14)	0.0127 (15)
O1	0.0614 (8)	0.0528 (7)	0.0533 (8)	0.0088 (6)	0.0310 (6)	0.0040 (6)
O2	0.0770 (11)	0.0842 (11)	0.0665 (10)	0.0217 (9)	0.0315 (8)	0.0249 (8)
O3	0.0500 (7)	0.0556 (8)	0.0513 (8)	−0.0048 (6)	0.0158 (6)	0.0033 (6)
O4	0.0642 (10)	0.0731 (11)	0.1270 (16)	−0.0078 (8)	0.0344 (10)	0.0250 (11)

### Geometric parameters (Å, °)

**Table:**

C1—O3	1.423 (2)	C7—H7	0.93
C1—O1	1.441 (2)	C8—N1	1.141 (3)
C1—C2	1.504 (3)	C9—O2	1.197 (3)
C1—H1	0.98	C9—O1	1.355 (2)
C2—C7	1.381 (3)	C9—C10	1.483 (3)
C2—C3	1.386 (3)	C10—H10A	0.96
C3—C4	1.384 (3)	C10—H10B	0.96
C3—H3	0.93	C10—H10C	0.96
C4—C5	1.381 (3)	C11—O4	1.196 (3)
C4—H4	0.93	C11—O3	1.362 (2)
C5—C6	1.384 (3)	C11—C12	1.482 (3)
C5—C8	1.451 (3)	C12—H12A	0.96
C6—C7	1.386 (3)	C12—H12B	0.96
C6—H6	0.93	C12—H12C	0.96
			
O3—C1—O1	105.26 (14)	C6—C7—H7	119.8
O3—C1—C2	108.62 (14)	N1—C8—C5	179.2 (3)
O1—C1—C2	109.80 (14)	O2—C9—O1	122.60 (18)
O3—C1—H1	111.0	O2—C9—C10	126.2 (2)
O1—C1—H1	111.0	O1—C9—C10	111.2 (2)
C2—C1—H1	111.0	C9—C10—H10A	109.5
C7—C2—C3	119.60 (18)	C9—C10—H10B	109.5
C7—C2—C1	121.08 (17)	H10A—C10—H10B	109.5
C3—C2—C1	119.32 (16)	C9—C10—H10C	109.5
C4—C3—C2	120.36 (19)	H10A—C10—H10C	109.5
C4—C3—H3	119.8	H10B—C10—H10C	109.5
C2—C3—H3	119.8	O4—C11—O3	122.2 (2)
C5—C4—C3	119.73 (19)	O4—C11—C12	126.4 (2)
C5—C4—H4	120.1	O3—C11—C12	111.37 (19)
C3—C4—H4	120.1	C11—C12—H12A	109.5
C4—C5—C6	120.28 (19)	C11—C12—H12B	109.5
C4—C5—C8	120.14 (19)	H12A—C12—H12B	109.5
C6—C5—C8	119.6 (2)	C11—C12—H12C	109.5
C5—C6—C7	119.7 (2)	H12A—C12—H12C	109.5
C5—C6—H6	120.1	H12B—C12—H12C	109.5
C7—C6—H6	120.1	C9—O1—C1	116.28 (15)
C2—C7—C6	120.30 (19)	C11—O3—C1	116.52 (15)
C2—C7—H7	119.8		

### Hydrogen-bond geometry (Å, °)

**Table:**

*D*—H···*A*	*D*—H	H···*A*	*D*···*A*	*D*—H···*A*
C1—H1···O2^i^	0.98	2.56	3.351 (3)	137

Symmetry codes: (i) *x*, −*y*+1/2, *z*−1/2.
